# *Plasmodium falciparum* malaria importation from Africa to China and its mortality: an analysis of driving factors

**DOI:** 10.1038/srep39524

**Published:** 2016-12-21

**Authors:** Shengjie Lai, Nicola A. Wardrop, Zhuojie Huang, Claudio Bosco, Junling Sun, Tomas Bird, Amy Wesolowski, Sheng Zhou, Qian Zhang, Canjun Zheng, Zhongjie Li, Andrew J. Tatem, Hongjie Yu

**Affiliations:** 1 WorldPop, Department of Geography and Environment, University of Southampton, Southampton SO17 1BJ, UK; 2 Division of Infectious Disease, Key Laboratory of Surveillance and Early–warning on Infectious Disease, Chinese Center for Disease Control and Prevention, Beijing 102206, China; 3 Flowminder Foundation, Stockholm, Sweden; 4 Department of Ecology and Evolutionary Biology, Princeton University, New Jersey, USA; 5 Center for Communicable Disease Dynamics, Harvard T. H. Chan School of Public Health, Boston, USA; 6 School of Public Health, Fudan University, Key Laboratory of Public Health Safety, Ministry of Education, Shanghai, China

## Abstract

*Plasmodium falciparum* malaria importation from Africa to China is rising with increasing Chinese overseas investment and international travel. Identifying networks and drivers of this phenomenon as well as the contributors to high case-fatality rate is a growing public health concern to enable efficient response. From 2011–2015, 8653 *P. falciparum* cases leading to 98 deaths (11.3 per 1000 cases) were imported from 41 sub-Saharan countries into China, with most cases (91.3%) occurring in labour-related Chinese travellers. Four strongly connected groupings of origin African countries with destination Chinese provinces were identified, and the number of imported cases was significantly associated with the volume of air passengers to China (P = 0.006), parasite prevalence in Africa (P < 0.001), and the amount of official development assistance from China (P < 0.001) with investment in resource extraction having the strongest relationship with parasite importation. Risk factors for deaths from imported cases were related to the capacity of malaria diagnosis and diverse socioeconomic factors. The spatial heterogeneity uncovered, principal drivers explored, and risk factors for mortality found in the rising rates of *P. falciparum* malaria importation to China can serve to refine malaria elimination strategies and the management of cases, and high risk groups and regions should be targeted.

The international spread of infectious diseases including *Plasmodium falciparum* malaria has been accelerated by increasing human mobility via air travel over recent decades^1^,^2^,^3^. With many countries moving towards national malaria elimination, global eradication has risen up the international agenda^4^,^5^. However, *P. falciparum* malaria importation from endemic regions and the threat of spreading drug resistance remains a problem for many eliminating or malaria-free countries due to the difficulty of diagnosis, substantial burden of treatment, relatively high mortality rates, and potential secondary local transmission^6^,^7^. The importation of malaria from Africa has been common over the past decades in non-endemic countries such as the UK and France, that have historical, language and cultural ties^8^,^9^, and certain demographic groups exhibit substantially higher infection rates, such travellers visiting friends and relatives in endemic countries^7^,^10^,^11^.

An emerging route of *P. falciparum* infection movements recently is from Africa to China by Chinese migrant workers^12^,^13^. This rise has been witnessed over the past decade, corresponding with increased investment and movement of workers from China^14^,^15^. Historically, malaria has been widespread in China, with an estimate of approximately 30 million cases occurred annually in the 1940s^16^. Since the initiation of National Malaria Elimination Action Plan (2010–2020) in May 2010, which aims to eliminate malaria by 2020, autochthonous malaria cases have numbered in the hundreds annually, and the spatial distribution of locally acquired *P. falciparum* malaria in China has been significantly narrowed to Yunnan and Hainan provinces^17^,^18^. However, imported *P. falciparum* malaria is on the rise, with a higher case-fatality rate reported than that for countries in north America and Europe^7^,^11^,^12^,^13^, leading to a threat to the health of travellers and challenges to the Chinese healthcare system, as well as increasing the potential for re-introduction and onward transmission in malaria-free receptive areas^12^,^13^,^18^,^19^,^20^.

Quantifying the connectivity and drivers of international movements of malaria across continents and its risk factors for mortality has significance for the management of imported cases in receiving areas and development of mitigation strategies^8^,^21^. For example, identifying which regions tend to import cases from certain parts of Africa more than others means that local health facilities can be aware of risks and make more rapid diagnoses to save lives, undertake proactive interventions, such as prophylaxis to high risk travellers, and tailoring public health awareness campaigns^22^,^23^. The majority of imported *P. falciparum* malaria cases in China have been reported in those with a history of labour-related travel to Africa^12^,^13^,^19^,^20^, and thus, identifying potential high risk groups of travellers and the factors driving these risks facilitates both the tailoring of interventions^24^,^25^ and an understanding of how these risks might impact future importation rates, supporting strategy design.

Despite the public health relevance, existing analyses of *P. falciparum* malaria connectivity and importation have only considered China in the context of neighbouring countries in Asia^22^,^23^, and the patterns and driving factors of *P. falciparum* malaria importation from sub-Saharan Africa (SSA) to China have not been addressed. We therefore sought to quantify the patterns of malaria importation to China from Africa and explore these as a function of key driving factors, including volume of air travellers, parasite prevalence in Africa, and investments from China. In addition, we examine the factors behind the high mortality rates in imported cases. Improved understanding of the importation phenomenon can enable the development of rational, evidence based interventions in China to reduce levels of importation, lower mortality rates and design strategies to limit onward transmission risks.

## Results

### Characteristics of *P. falciparum* cases imported from SSA to China

**Figure 1:**
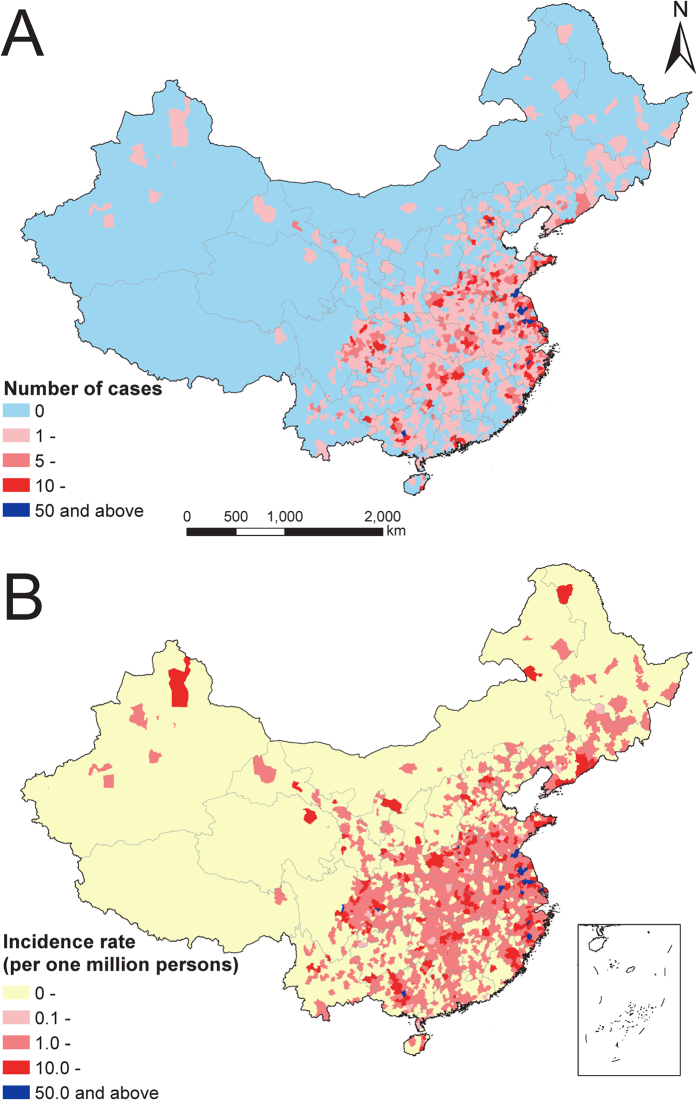
The distribution of imported *P. falciparum* malaria cases by county in mainland China, 2011–2015. (**A**) Number of cases in mainland China (31 provinces). (**B**) Overall 5-year incidence rate per one million persons by county. The map was created using ArcGIS 10.3 (www.esri.com/software/arcgis).

From 2011 to 2015, a total of 8,653 *P. falciparum* malaria cases recorded in China were imported from SSA, with an overall 5-year incidence rate of 6.5 cases per one million persons. The median age of patients was 40 years (Interquartile range [IQR] 31–46) with a strong male predominance (27.7:1) (Supplementary Fig. S1 and Table S1). The African cases (258) only accounted for 3.0% of all imported cases, and most cases were Chinese (97.0%) with a median duration of stay in SSA of 317 days (IQR 168–496), and 91.3% of cases were Chinese migrant workers. The distribution of imported cases in China varied, with the highest density in the counties of Guangxi province of southern China, Jiangsu and Anhui provinces in eastern China, and Sichuan provinces in western China (≥50 cases per one million persons) (Fig. 1 and Supplementary Fig. S2).

### Connectivity and community of *P. falciparum* importation

**Figure 2:**
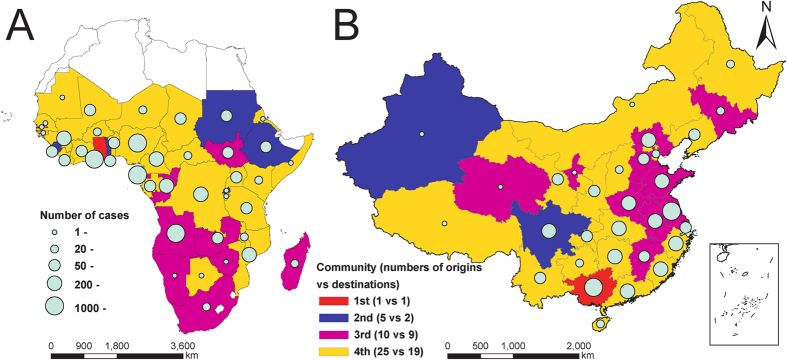
Four communities of origin-destination networks of *P. falciparum* malaria importation from SSA to mainland China. (**A**) Origins (41 countries) in sub-Saharan Africa. (**B**) Destinations (31 provinces) in mainland China. The origin countries linked to a median of 18 provinces (IQR 8–23) in mainland China, with Angola was the most connective country linking to 30 provinces. Conversely, destination provinces in mainland China linked to a median of 21 countries (IQR 13–26), with Guangdong province was the most connective destination receiving cases from 34 countries. The score of modularity is 0.219 with a resolution of 0.9, and the list of origin-destination communities is provided in Supplementary Table S2. The map was created using ArcGIS 10.3 (www.esri.com/software/arcgis).

41 sub-Saharan countries exported *P. falciparum* into China, with Ghana (20.0%, 1734 cases), Angola (15.4%, 1333), and Nigeria (12.4%, 1076) being the top three origins. All 31 provinces in mainland China reported imported cases, with Guangxi (17.9%, 1548), Jiangsu (15.7%, 1355), and Henan (7.8%, 677) provinces as top three destinations (Supplementary Fig. S3). The median number of imported cases was 4 cases (IQR 2–11) for each origin-destination pair, and four distinct communities were identified in this imported malaria flow matrix (Fig. 2 and Supplementary Table S2). The first community included Ghana and Guangxi province: this link constituted the largest number of malaria case importations between SSA and China (1311 cases). The second community included five countries (Sudan, Ethiopia, Sierra Leone, Togo, and Rwanda) in Africa and two provinces (Xinjiang and Sichuan) in China; the third had ten countries with most in southern Africa and nine provinces with most in eastern China; others constituted the fourth community.

### Driving factors of the importation phenomena

**Figure 3:**
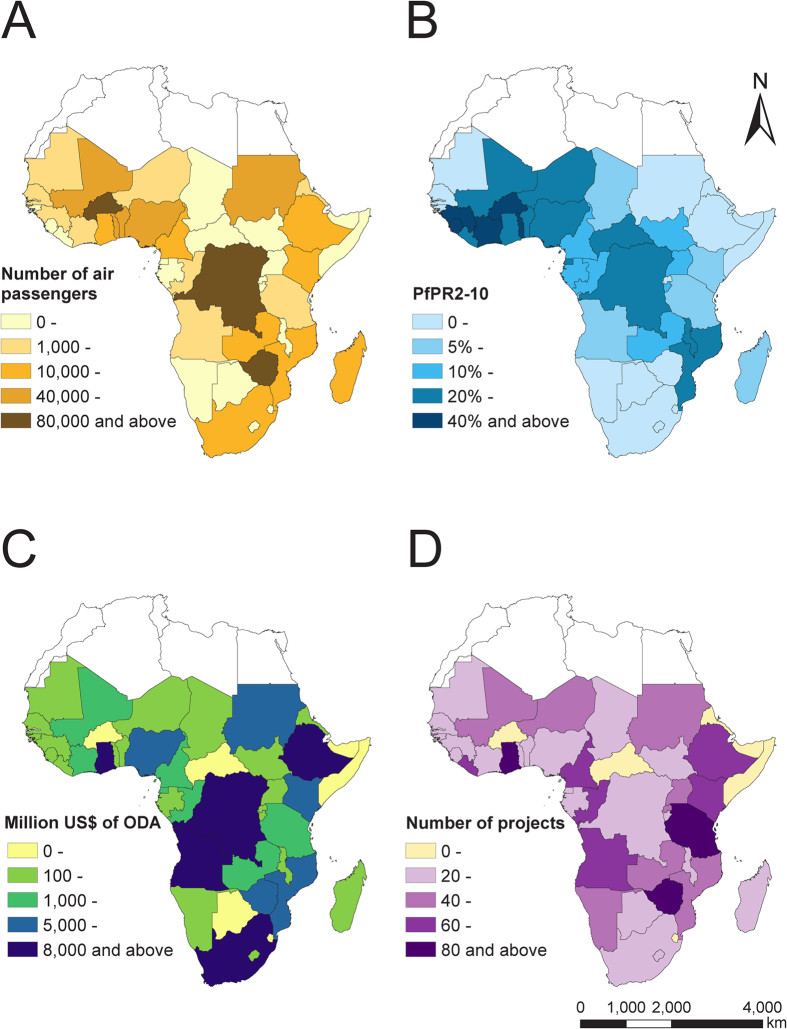
The distribution of air travellers from sub-Saharan Africa to China, malaria risk in Africa, and official development assistance from China by country. (**A**) Number of air passengers from sub-Saharan countries to China. (**B**) Mean of *P. falciparum* malaria prevalence (*PfPR*_*2–10*_) by country from 2010 to 2015. (**C**) The total amount of official development assistance (ODA) from China into sub-Saharan countries in 2006–2013. The monetary amount was deflated from reported currency to US. Dollars in 2011. (**D**) The numbers of projects of ODA from China into sub-Saharan countries in 2006–2013. The map was created using ArcGIS 10.3 (www.esri.com/software/arcgis).

According to *Spearman*’s correlation coefficient, the number of *P. falciparum* cases exported from each SSA country to China was significantly associated with the volume of air passengers (median 2,080 persons, IQR 405–34,600; ρ = 0.425, P = 0.006), *PfPR*_2–10_ (10.7%, 2.5%–30.6%; ρ = 0.639, P < 0.001), and total official development assistance (ODA) from China (610.4 million US $, 219.7–4,654.0; ρ = 0.679, P < 0.001) (Fig. 3 and Supplementary Table S3). By sector of ODA, significant correlations were found between the numbers of cases and investment in natural resource extraction (P < 0.001), infrastructure (P = 0.002), health (P=0.001), education (P = 0.054), multi-sector (P = 0.034), and “other” (P = 0.011) (Supplementary Fig. S4 and Table S3). Adjusting for the volume of air travellers and *PfPR*_2–10_, the quasi-Poisson regression model fitted by total ODA explained 65.9% (IQR 63.3–69.8%) of the deviance of the number of cases in the training dataset, and 57.7% (23.7–79.7%) in cross-validation. In the model, the total ODA and *PfPR*_2–10_ were positively correlated with numbers of cases with coefficients of 1.0 (IQR 0.96–1.13) and 0.70 (IQR 0.64–0.8), while volume of travellers had a negative coefficient of −0.45 (IQR −0.54 to −0.41) (Supplementary Fig. S5 and Table S4).

### Risk factors for deaths in imported cases

The case-fatality rate was 11.3 per 1,000 cases (98 deaths in 8653 *P. falciparum* imported cases), and potential mortality risk factors with adjusted odds ratios (OR) and 95% confidence interval (CI) are summarised in Supplementary Table S5. The significant mortality risk factors identified by multivariable logistic regression model were mainly related to the capacity of malaria diagnosis and socio-economic status of cases, including the first-visit health institution at township level or lower (OR 2.6, 95%CI 1.5–4.2), >3 days between onset and diagnosis (2.2, 1.3–4.0), onset in January and February (2.3, 1.4–3.7), age > 50 years (2.4, 1.2–4.4), and cases from provinces with GDP per capita < =12,000 US$ (1.9, 1.0–3.9), low education (1.8, 1.0–3.3), and cases from communities 2^nd^ to 4^th^ identified by network modularity analysis above (Table 1).

## Discussion

The data and analyses presented here highlight an emerging route of infectious disease importation for *P. falciparum* malaria from Africa to China over the last half decade. The level of importation from SSA to China is contrary to the previous perception that China’s imported malaria mainly originates in neighbouring countries of southeast Asia^22^,^23^. Volume of air travel from African countries to China, prevalence of malaria in origin countries and Chinese investments in African countries were all associated with the number and distribution of imported cases, with diverse risk factors for mortality found. Malaria elimination strategies in China should account for these trends and challenges, and malaria diagnosis efforts and healthcare should target high risk groups and regions to reduce case-fatality rates.

Although Africa has been found to be the main sources of imported malaria infections in China since 2010 in previous descriptive studies^12^,^13^, the heterogeneity of communities mapped between origin SSA countries and destination Chinese provinces (e.g. the strongest linkage was between Ghana and Guangxi province) in this analysis reflects the variability of connections and driving factors that exist. The presence of four distinct communities relates to patterns in investments and volume of workers moving between specific provinces in China and specific countries in SSA^19^,^20^. The strong origin-destination importation pair between Ghana and Guangxi Province in China is likely caused by the large number of migrant workers from Shanglin county in Guangxi to Ghana, with >10,000 inhabitants travelling abroad to conduct gold mining work in Ghana since 2006. Moreover, Ghana began to strictly regulate the gold mining industry in late April 2013, which forced many gold miners to return to China within a short period, and more than 800 *P. falciparum* cases were found through screening in Shanglin during May–August 2013^19^.

The magnitude of importation from sub-Saharan countries was significantly correlated with the volume of air passengers (although the coefficient was negative in the regression model, after inclusion of other covariates), *PfPR*_2–10_, and ODA. Recent ODA investments (in particular natural resource extraction projects) by Chinese companies^14^, mainly state-owned enterprises, has led to growth in the number of migrant Chinese labourers in SSA, and those engaged in outdoor activities are at particularly high risk for malaria infection^12^,^13^,^19^. The findings in this analysis also echo a previous retrospective study in Jiangsu Province of China covering the period of 2001–2011, which found that the increase of imported malaria cases was associated with the growth of investment and the increasing number of Chinese labourers to Africa from Jiangsu^20^. Furthermore, the lack of acquired immunity in Chinese citizens increases their vulnerability to *P. falciparum*, and their long stays (median 317 days) also increase the risk of exposure. Additionally, the labourers are generally poorly educated and lack awareness of the risk of malaria and personal protection against mosquito bites, and the majority of workers do not obtain anti-malarial medication prior to overseas travel^13^,^26^. Thus, targeting these workers, especially employees in resource extraction related businesses, with malaria awareness messages and provision of prophylaxis is likely to be an efficient strategy.

We found that socio-economic factors were associated with case fatality in imported *P. falciparum* cases, with a higher fatality rate in those with a lower education level or from the provinces with lower GDP per capita. Most cases were migrant workers from less developed regions in China, who might lack knowledge of malaria prevention, experience poor healthcare accessibility, and delay treatment seeking behaviour^27^. Care-seeking from hospitals at county or higher levels in the first instance might reduce the risk of fatality as the low capacity for malaria diagnosis at township or lower levels, especially in non-endemic areas, is likely to contribute to high fatality rates. Moreover, we found higher case fatality rates occurred in imported cases during January and February, which normally covers the Chinese New Year holidays. In this period, delayed presentation to medical services, lower levels of hospital staffing over the holiday period, and delays in communication of malaria diagnosis from laboratory to physician may be contributory factors, and other possibilities include initial misdiagnosis of a febrile illness as influenza-like illness in the winter-spring epidemic season^7^,^28^. Additionally, the fatal cases had a shorter median length of stay, which might relate to the severity of infection, healthcare-seeking behaviours, malaria prevention measures, individual immunity and the previous history of malaria, among other factors. The results here therefore point towards the need to increase malaria diagnostic capacity in rural, low-level facilities, improve staffing over holidays, and provide health messages to encourage seeking treatment if ill after travel to Africa.

There is heterogeneity in destination provinces for imported *P. falciparum* cases, with a high density in the Yangzi-river delta areas, the centre of Guangxi, and the east of Sichuan province (Supplementary Fig. S2). Further, Yunnan and Hainan provinces, where autochthonous transmission of *P. falciparum* malaria occurs, also have imported cases^17^, which poses a challenge for national malaria elimination. *Anopheles* that can transmit malaria are found in both malaria-endemic areas of China and in areas where malaria has already been eliminated^29^, and climate change may change habitats and predominant vector species^30^. Therefore, malaria importation is a continual and evolving threat which may undermine elimination efforts in China, especially in areas historically endemic for *P. falciparum* malaria and areas with high transmission suitability, where the vector may be present. It also becomes increasingly important to ensure that surveillance systems capture data on imported cases, and designing intervention strategies that target areas of high importation and have had past transmission. Future work should be focussed on estimating transmission potential in areas of high parasite importation.

There are some limitations to this study. First, the case data used were collected from passive public health surveillance. The data quality may be influenced by the key steps in surveillance including reporting methods, availability of health facilities and laboratory diagnostics, under reporting, completeness and accuracy of data over the years. Because of the classification of imported cases before 2010, previous history of malaria infections and number of previous trips were unavailable for this analysis, thus, the assessment of a longer term trend and the effects of previous malaria infections and travel patterns were not conducted in this study. Second, the economic data only include Chinese official financing without data of private-sector investments, and the original data source is likely of variable accuracy. Third, we cannot capture the movement and detailed location of air travellers: we used modelled travel data from 2010, although the relative patterns of air travel might have changed from 2010 to 2015. Moreover, our importation calculations are non-seasonal, constrained by the available malaria prevalence estimates, air travel and economic data.

Strategies for targeting *P. falciparum* importation from SSA related to Chinese investment-related travel will likely be different from those that concentrate on local transmission in China. The evidence of this study, by mapping these emerging routes and defining drivers of parasite dispersal by human carriers, suggests that national malaria elimination programs should account for labour travel-mediated malaria spread. Interventions for reducing this importation pathway should communicate risks to travellers to alter their behaviours and improve regional capacity for diagnosis and treatment to prevent death. Strong surveillance systems need to be maintained to sustain the status of elimination in malaria-free regions by monitoring the risk of importation and the transmission potential in risk areas and form a cornerstone of post-2015 elimination strategies in China^31^,^32^.

## Methods

A comprehensive database of individual malaria cases imported from SSA to China between 2011 and 2015 was constructed. We also compiled covariate datasets covering Chinese investment in African countries, air travel volumes between China and African countries and malaria endemicity across SSA. We used these data to describe *P. falciparum* malaria importation networks from SSA to China and to examine driving factors for malaria importation and its mortality. Here we describe the data assembly and analysis, with further details provided in supplemental information.

### Database compilation

We compiled individual *P. falciparum* malaria case records reported during 2011–2015 in mainland China (Supplementary Table S6): cases were diagnosed according to the unified national diagnostic criteria, including clinically diagnosed and laboratory-confirmed cases^33^. The travel histories of each imported malaria case were investigated by local health departments using a standard questionnaire from the Technical Scheme of China Malaria Elimination (2011) to record the location of suspected infection acquisition^34^. A malaria patient was classified as an imported case if the individual travelled to a malaria-endemic country within the month prior to diagnosis, and the last country visited was taken as the origin of infection^34^.

To examine driving factors for malaria importation from SSA to China, data describing malaria endemicity, population movements and investments were collated. *P. falciparum* parasite prevalence estimates in 2–10 year-olds (*Pf*PR_2–10_) across Africa from 2010 to 2015, were obtained from the Malaria Atlas Project^5^, as an indicator of risk of infection for Chinese travellers lacking acquired immunity. Mean *Pf*PR_2–10_ was calculated for each country after weighting by population density (using data acquired from the WorldPop project; www.worldpop.org)^35^. The volume of air passengers from SSA to China, predicted based primarily on publicly available datasets from 2010 under a generalized linear model framework in previous studies, was acquired^23^,^35^. Travel data for direct, one-stop and two-stop flights were aggregated with the assumption that the air travel patterns, in terms of relative strength of connections from SSA to mainland China, were consistent during 2010–2015.

Investment data relating to Chinese ODA to SSA between 2006 and 2013 were obtained from AidData (china.aiddata.org), which collates information using a systematic and replicable approach to generate open-source, project-level data^36^. Private-sector data are not available, and we make the assumption that investment from official and private sectors from China will follow the same patterns. The ODA flows used in this study include grants, technical assistance, concessional and non-concessional loans, debt relief, export credits, and other financial instruments. We further grouped the ODA data into six sectors: resource extraction (energy, mining and agriculture), infrastructure (transport, communications and utilities), health, education, multi-sector, and “other”. The monetary amount was deflated from reported currency to 2011 US Dollars (Supplementary Table S7).

### Statistical analyses

The epidemiologic characteristics of imported *P. falciparum* cases were summarized, and the crude 5-year incidence rates were estimated as the total number of cases divided by the population at 2010 year-end from census data in China. Based on the travel history of each case, the connectivity between SSA countries (origins) exporting *P. falciparum* and Chinese provinces (destinations) were defined by network modularity analysis (Supplementary Table S2)^37^. By mapping communities on the importation network, we aimed to identify groups of origin-destination pairs that show strong links in terms of movements of infected travellers.

The *Spearman’s* rank correlation coefficient (ρ)^38^ was employed to test the relationship between the counts of imported cases and covariates (investment value from China, volume of air travellers from origin country to China, and *Pf*PR_2–10_ in origin country) by sub-Saharan country. To explore the impact of ODA in each investment category on malaria importation from SSA, a generalized linear regression model was constructed, adjusting for parasite prevalence at origin and volume of air travellers from origin country to China. The Quasi-Poisson distribution was used due to over-dispersion (the counts of imported cases were positively skewed and subject to outliers)^39^. Model validation was performed using cross-validation with repeated random sub-sampling, iterated 1000 times (80% training and 20% testing). The strength of relationships was examined using R-square (R^2^).

To examine potential risk factors (Table 1) for case fatality we used bivariate and multivariate logistic regression, with mortality as a binary outcome from all imported *P. falciparum* malaria cases from SSA. All covariates found to be significantly associated with mortality (P < 0.05) in univariate analysis were included in a multivariable logistic regression model, controlling for confounders age, sex and nationality. Version 3.2.3 of the *R* statistical software (R Foundation for Statistical Computing, Vienna, Austria)^40^ was used to conduct statistical analyses, and the ArcGIS 10.3 (ESRI, Redlands, CA, USA) was used to plot the geographical distribution of cases and conduct spatial analyses.

### Ethical approval

It was determined by the National Health and Family Planning Commission, China that the collection of malaria case reports was part of continuing public health surveillance of a notifiable infectious disease and was exempt from institutional review board assessment. All other data were obtained from publicly available data sources. This work forms part of the study for the role of population mobility on the mosquito-borne diseases dynamics of China, for which ethical clearance was granted by the institutional review board of the University of Southampton, UK (No. 18152). All data were supplied and analysed in an anonymous format, without access to personal identifying information.

## Additional Information

**How to cite this article**: Lai, S. *et al. Plasmodium falciparum* malaria importation from Africa to China and its mortality: an analysis of driving factors. *Sci. Rep.* **6**, 39524; doi: 10.1038/srep39524 (2016).

**Publisher's note:** Springer Nature remains neutral with regard to jurisdictional claims in published maps and institutional affiliations.

## Supplementary Material

Supplementary Information

## Figures and Tables

**Table 1:** Factors associated with risk of death in *P. falciparum* malaria cases imported from sub-Saharan countries to mainland China, 2011–2015.

Factor	OR (95% CI)	P value
Gender - Male	2.1 (0.4, 37.4)	0.469
Age >50 years	2.4 (1.2, 4.4)	0.009
Nationality - Chinese	1.5 (0.3, 27.8)	0.675
Education - Primary or lower	1.8 (1.0, 3.3)	0.055
Community 2^nd^ of origin-destination	6.2 (1.5, 41.2)	0.022
Community 3^rd^ of origin-destination	3.8 (1.1, 24.6)	0.072
Community 4^th^ of origin-destination	5.7 (1.6, 35.7)	0.020
GDP per capita by province < = 12000 US$	1.9 (1.0, 3.9)	0.048
Onset in January and February	2.3 (1.4, 3.7)	0.001
Duration from onset to diagnosis > 3 days	2.2 (1.3, 4.0)	0.006
*PfPR*_2–10_ in origins of SSA in 2010–2015 < = 20%	1.5 (0.9, 2.4)	0.140
First-visit health institution at township level or lower	2.6 (1.5, 4.2)	<0.001

Note: OR: odds ratio; CI: confidence interval. All potential risk factors (Supplementary Table S5) statistically associated with mortality (P < 0.05) found in univariate analysis and potential confounders (factors age, sex, and nationality) were introduced into multivariable logistic regression model to explore the significant risk factors. A total of 7,025 cases (81.2% of 8,653 cases) with complete data were included in this model. Communities of origin-destination were identified by network modularity analysis.
