# Supplementary material for: Plasmodium falciparum malaria importation from Africa to China and its mortality: an analysis of driving factors
Source: Sci Rep. 2016 Dec 21;6:39524. doi: 10.1038/srep39524 (PMC5175130; doi:10.1038/srep39524)
Supplement: Supplementary Information [file srep39524-s1.pdf]

Supplemental information for

Plasmodium falciparum malaria importation from Africa to China and its mortality: an analysis of driving factors

Shengjie Lai, Nicola A. Wardrop, Zhuojie Huang, Claudio Bosco, Junling Sun, Tomas Bird, Amy Wesolowski, Sheng Zhou, Qian Zhang, Canjun Zheng, Zhongjie Li, Andrew J. Tatem & Hongjie Yu

This document includes:

Database compilation

Malaria case definition and data sources

P. falciparum malaria prevalence data in Africa

Air travel flow data from Africa to China

Official financial flow data from China to Sub-Saharan Africa

Other data

Data analyses

Mapping the distribution of P. falciparum cases in China

Identifying communities of malaria importation networks

Exploring driving factors of malaria importation

Defining risk factors of mortality in imported P. falciparum

Supplementary Tables S1-S7

Supplementary Figures S1-S6

References

Database compilation

Malaria case definition and data sources

This study included all records of *P. falciparum* malaria cases during 2011–2015 imported from sub-Saharan Africa (SSA) to all provinces in mainland China, which includes 22 provinces, four municipalities, and five autonomous regions. Laboratory-confirmed malaria cases refer to patients with any positive result in the following diagnostic tests: malaria parasites confirmed by microscopy, rapid diagnostic tests, or polymerase chain reaction tests. All other cases with malaria-like symptoms and a history of travelling to a malaria endemic area in malaria transmission season, or a history of blood transfusion in past 2 weeks, but without positive laboratory test results, were identified as clinically diagnosed malaria cases.[1]

Two data sources of individual *P. falciparum* cases were used in this study. One includes demographic information, date of onset, date of diagnosis, date of reporting, and reporting institute, obtained from the National Notifiable Infectious Disease Reporting Information System (NIDRIS). The other consists of epidemiological information on the course of diagnosis, history of travel, treatment, classification (autochthonous or imported case), collated from Malaria Enhanced Surveillance Information System (MESIS).[2] The MESIS was developed in 2010 as an effort of National Malaria Elimination Action Plan, and the same individual case recorded in NIDRIS and MESIS is linked by unique code. The cases have been checked in NIDRIS and MESIS by the variables of name, identity number and gender to avoid the duplicative reporting, and only case with new infection is reported in the surveillance systems.

P. falciparum malaria prevalence data in Africa

To assess the impact of malaria endemicity on the importation, parasite rate of *P. falciparum* in 2- to 10-year-olds (*Pf*PR$_{2\text{-}10}$) in Africa from 2010 to 2015 were obtained from the Malaria Atlas Project (www.map.ox.ac.uk). The methods behind their construction are presented in Bhatt et al.[3] In brief, a large database of malaria infection prevalence survey points (n= 27,573) were combined with intervention data, and environmental and

sociodemographic covariates within a spatiotemporal Bayesian geostatistical model to map the yearly prevalence of *P. falciparum* in children age 2-up-to-10 at 5 × 5 km resolution across sub-Saharan Africa from 2000 to 2015. A map of mean $Pf\mathrm{PR}_{2-10}$ during 2010-2015 was created and weighted by the averaged population density in 2010 and 2015 of each country which were obtained from the WorldPop Project (www.worldpop.org). Then an average value of $Pf\mathrm{PR}_{2-10}$ was aggregated for each country and used in the statistical analysis of this study. Because most Chinese cases had a long stay in SSA (median: 317 days) and might be infected in previous year, the data of $Pf\mathrm{PR}_{2-10}$ in 2010 were also included in this analysis.

Air travel flow data from Africa to China

The flow matrix from SSA to mainland China were obtained from an open-access modelled passenger flow matrix of the global air networks for the Vector-borne Disease Airline Importation Risk Tool (www.vbd-air.com). This dataset was modelled based primarily on publicly available datasets in 2010 under a generalized linear model framework by Huang et al.[4,5] To construct the matrix, topological characteristics of the air travel network, city population, and local area GDP, amongst others, were utilized as covariates, and the actual travel volumes were extracted and assembled for training and validation. A log linear model controlling for random effects on origin, destination and the airport hierarchy was then built to predict passenger flows on the network. The model outperformed existing air travel passenger flow models in terms of prediction accuracy.[4,5]

The vector imported was not taken into account due to few direct flight from Africa to China, and air travel likely plays a much more significant role in moving the vector-borne disease (via infected passengers) than in moving the vector itself. Additionally, as the long geographic distance from Africa to China and few travel by road and water, only air travel data were included in this study. The number of air passengers of direct, one-stop and two stops flights from airports in SSA to airports in China were aggregated for each sub-Saharan country.

Official financial flow data from China to Sub-Saharan Africa

To define the impact of investment from China on malaria importation, the dataset of Official Development Assistance (ODA) from the People's Republic of China to Africa between 2006 and 2013 were obtained from the AidData Project (china.aiddata.org). China is fundamentally changing the development finance landscape; however, there are few statistical data reported officially, and China does not participate in existing global reporting systems, such as the Organization for Economic Cooperation and Development's (OECD) Creditor Reporting System and the International Aid Transparency Initiative. Therefore, the AidData has collated official financing from China to Africa using a systematic and replicable approach to generate open-source, project-level data of international development finance.[6] The official development finance flows included grants, technical assistance, concessional and non-concessional loans, debt relief, export credits, and other financial instruments.

The methodology of data collation in the AidData was divided into two stages.[7] In Stage One, researchers followed a step-by-step guide to identify potential sources of project-level information from the Factiva (global.factiva.com), publicly available official data and documentation from donor/creditor and recipient/borrower governments. Projects were given unique identification numbers as they were discovered and entered into the database. All available project information was input into database, including links to the underlying source documentation. The primary objective of Stage One was to identify and record as many potential projects as possible. In Stage Two, each project record created in Stage One was carefully assessed and enhanced through targeted Google and Google Scholar searches, using a corpus of relevant search terms developed by the AidData. Researchers compiled and triangulated information from these diverse sources in order to accurately populate as project fields as possible. This process of stage-two refinement was designed to minimize reliance upon "sole-sourced" records.

The unit of record in dataset is the "project", broadly defined, which is a discrete transfer of goods, services or cash. The pledges or cancelled/suspended projects were not included in the aggregate amounts of Chinese official financing. The monetary amount was deflated

from reported currency to 2011 U.S. Dollars. In addition, the duration of each project is unavailable, and normally projects need several years to conduct, we therefore included the data since 2006, five years before the study period of 2011-2015, with the assumption that each project will last 5 years.

Other data

To quantify the impact of social-economic factors on the risk of mortality in malaria cases, we collected the data of gross domestic product (GDP) per capita of each province in mainland China in 2015,[8,9] referring to the development level of provinces where malaria cases came from (address of onset).

Data analyses

Mapping the distribution of *P. falciparum* cases in China

To visualize the geographic distribution of imported cases in China, using the coordinate of each case's location of illness onset in China (Supplementary Fig. S2), we fitted a smoothly tapered density surface to the coordinate of individual cases at a magnitude-per-unit area based on the quartic kernel function described by Silverman.[10] Conceptually, a smoothly curved surface is fitted over each point, and the surface value is highest at the location of the point and diminishes with increasing distance from the point, reaching zero at the search radius distance from the point. The algorithm used to determine the search radius, also known as the bandwidth is:

$$SearchRadius = 0.9 \times min\left(SD, \sqrt{\frac{1}{\ln(2)}} D_m\right) n^{-0.2}$$

where D_m is the median distance from the mean center for all points, SD is the standard distance, and n is the number of points. The density at each output raster cell is calculated by adding the values of all the kernel surfaces where they overlay the raster cell center. The output density surface was compiled at a spatial resolution of 0.083333 decimal degrees per pixel (approx. 10km at the equator).

Identifying communities of malaria importation networks

The communities of malaria importation networks were detected by modularity analysis. The modularity of a partition is a scalar value between -1 and 1 that measures the density of links inside communities as compared to links between communities.[11] The score of modularity is defined by Newman and Girvan[12] as

$$Q = \frac{1}{2m}\sum_{i,j}\left[A_{ij} - \frac{k_i k_j}{2m}\right]\delta(c_i, c_j)$$

where A_{ij} represents the weight of the edge between i and j, $k_i = \sum_j A_{ij}$ is the sum of the weights of the edges attached to vertex i, c_i is the community to which vertex i is assigned, the δ-function $\delta(u, v)$ is 1 if $u = v$ and 0 otherwise and $m = \frac{1}{2}\sum_{ij} A_{ij}$.

Networks with high modularity have dense connections between the nodes within communities but sparse connections between nodes in different communities. We divided the *P. falciparum* importation networks into communities of densely connected the origins (nodes) of sub-Saharan countries and the destinations (nodes) of provinces in China. The resolution of modularity was set as 0.9 to generate suitable number of communities in the networks (Supplementary Figs. 3 and 4).[13]

Additionally, Ghana in 2013 began to strictly regulate the gold mining industry, which forced many Chinese gold miners returning to hometowns in Guangxi province within a short time, and a substantial proportion of them (21.6%) infected with malaria.[14] To account for this special event, we therefore exploited the communities of networks again after removing the cases of Ghana-Guangxi pair in January - October, 2013 (N = 1,057) during this special event of. We found that the remained data formed similar three communities (Supplementary Fig. S6) as the original data except for Ghana-Guangxi community (Supplementary Fig. S3).

Exploring driving factors of malaria importation

The relations between the number of cases and covariates were explored by the *Spearman*'s rank correlation coefficient (ρ), which is a nonparametric measure of statistical dependence between the ranking of two variables.[15] The *Spearman*'s coefficient ρ assesses how well the relationship (whether linear or not) between two variables can be described using a monotonic function, while *Pearson*'s correlation only assesses linear relationships.[16] The *Spearman* correlation between two variables is equal to the *Pearson* correlation between the rank values of those two variables, and if there are no repeated data values, a perfect *Spearman* correlation of 1 or −1 occurs when each of the variables is a perfect monotone function of the other.[17] Because the relationship of two variables without transformation might be linear or not in this study, *Spearman*'s correlation ρ was explored here.

Since the counts of cases tend to be positively skewed and subject to outliers, a generalized linear model with quasi-Poisson distribution function[18] was constructed to fit the numbers of cases from each sub-Saharan country with covariates. The quasi-Poisson function differs from the Poisson function only in that the dispersion parameter is not fixed at one, it therefore can model over-dispersion for count data of occurrences in fixed amount of time/space.[19] To understand the impact of investments, the models were fitted by each of the aggregated ODA covariates, while adjusting for *Pf*$PR_{2\text{-}10}$ and number of air passengers. To make full use of the dataset, the number of imported cases for each origin was adjusted by adding one before logarithmic transform to eradicate zero values.[20] Moreover, to compare model's coefficients of covariates that were measured on different scales, Z-scores standardization was applied for covariates.[21] Z-scores are expressed in terms of standard deviations from their means with a distribution of a mean of 0 and a standard deviation of 1. The formula of Z-scores is

$$z = \frac{x - \mu}{\sigma}$$

where x is an observation of a covariate, μ is the mean of observations of this covariate, while σ *is* the standard deviation. A repeated random sub sampling validation was

conducted, with 80% observations were randomly sampled as train set to build the models, and remaining 20% observations as validation set. Due to the low number of aggregated data (41 observations), a high number of iterations (1000 times) was applied to fully explore the dataset for avoiding the outliers have a too big effect on the final results in validation. The strength of relationships was examined by the median of the R-square (R^2) calculated for each of the iterations, and the coefficients of models with highest median of R^2 were plotted.

Defining risk factors of mortality in imported *P. falciparum*

Bivariate analysis and multivariable logistic regression were conducted, with odds ratios (OR), 95% confident internals (CI), and a significance level of α=0.05, to examine potential risk factors for case fatality, by comparing deaths from *P. falciparum* malaria with the denominator of all imported *P. falciparum* malaria cases from SSA to mainland China. Potential risk factors for death from imported falciparum malaria were studied by univariate analysis with unadjusted and adjusted OR. The biologically likely potential confounding factors, age, sex, and nationality were included as potential confounding factors for adjusted OR. Additionally, all risk factors found to be associated with mortality with a probability value P<0.05 in univariate analysis and potential confounding factors (age, sex, and nationality) were introduced into a multivariable logistic regression model to explore the significant risk factors.

Supplementary Tables

Supplementary Table S1. Characteristics of *P. falciparum* malaria cases imported from SSA to mainland China, 2011-2015.

Characteristics	Total (n=8,653)	Non-fatal cases (n=8,555)	Fatal cases (n=98)
Type of diagnosis			
Laboratory-confirmed	8,530 (98.6%)	8,433 (98.6%)	97 (99.0%)
Clinically diagnosed	123 (1.4%)	122 (1.4%)	1 (1.0%)
Sex			
Male	8,351 (96.5%)	8,259 (96.5%)	92 (93.9%)
Female	302 (3.5%)	296 (3.5%)	6 (6.1%)
Age			
Median (yrs, IQR)	40.0 (31.0, 46.0)	40.0 (31.0, 46.0)	44.2 (36.4, 49.5)
Age group			
0-4	7 (0.1%)	7 (0.1%)	0 (0)
5-14	6 (0.1%)	6 (0.1%)	0 (0)
15-24	688 (8.0%)	685 (8.0%)	3 (3.1%)
25-34	2,275 (26.3%)	2,257 (26.4%)	18 (18.4%)
35-44	3,022 (34.9%)	2,993 (35.0%)	29 (29.6%)
45-54	2,324 (26.9%)	2,283 (26.7%)	41 (41.8%)
55-64	307 (3.4%)	302 (3.4%)	5 (5.1%)
65 and above	24 (0.3%)	22 (0.3%)	2 (2.0%)
Nationality			
Chinese	8,395 (97.0%)	8,298 (97.0%)	97 (99.0%)
Foreigner	258 (3.0%)	257 (3.0%)	1 (1.0%)
Education			
Illiteracy	516 (6.0%)	504 (5.9%)	12 (12.2%)
Primary	880 (10.2%)	862 (10.1%)	18 (18.4%)
Junior secondary	4,091 (47.3%)	4,061 (47.5%)	30 (30.7%)
Senior secondary	1,276 (14.6%)	1,257 (14.7%)	19 (19.4%)
Higher education	1,244 (14.4%)	1,232 (14.4%)	12 (12.2%)

Characteristics	Total (n=8,653)	Non-fatal cases (n=8,555)	Fatal cases (n=98)
Unknown	646 (7.5%)	639 (7.4%)	7 (7.1%)
Travel purpose			
Labour	7,770 (89.8%)	7,679 (89.8%)	91 (92.9%)
Other	738 (8.5%)	731 (8.5%)	7 (7.1%)
Unknown	145 (1.7%)	145 (1.7%)	0 (0)
Hospitalization			
Yes	5,525 (63.9%)	5,441 (63.6%)	84 (85.7%)
No	2,894 (33.4%)	2,884 (33.7%)	10 (10.2%)
Unknown	234 (2.7%)	230 (2.7%)	4 (4.1%)
Year of onset			
2011	1,057 (12.2%)	1,033 (12.1%)	24 (24.5%)
2012	1,197 (13.8%)	1,184 (13.8%)	13 (13.3%)
2013	2,724 (31.5%)	2,703 (31.6%)	21 (21.4%)
2014	1,779 (20.6%)	1,757 (20.5%)	22 (22.4%)
2015	1,896 (21.9%)	1,878 (22.0%)	18 (18.4%)
Month of onset			
January	786 (9.1%)	763 (8.9%)	23 (23.5%)
February	533 (6.2%)	526 (6.1%)	7 (7.1%)
March	469 (5.4%)	459 (5.4%)	10 (10.2%)
April	641 (7.4%)	638 (7.5%)	3 (3.1%)
May	817 (9.4%)	805 (9.4%)	12 (12.2%)
June	1,305 (15.1%)	1,295 (15.1%)	10 (10.2%)
July	1,047 (12.1%)	1,042 (12.2%)	5 (5.1%)
August	635 (7.3%)	628 (7.3%)	7 (7.1%)
September	618 (7.1%)	615 (7.2%)	3 (3.1%)
October	619 (7.2%)	613 (7.2%)	6 (6.1%)
November	548 (6.3%)	543 (6.3%)	5 (5.1%)
December	635 (7.3%)	628 (7.3%)	7 (7.1%)
Median of time (days, IQR)			
From illness onset to diagnosis	3.0 (1.4, 6.0)	3.0 (1.4, 6.0)	6.3 (3.6, 7.8)
From diagnosis to report	0.1 (0.02, 0.7)	0.1 (0.02, 0.7)	0.2 (0.01, 0.7)

Characteristics	Total (n=8,653)	Non-fatal cases (n=8,555)	Fatal cases (n=98)
From illness onset to report	3.3 (1.6, 5.7)	3.0 (1.5, 5.7)	6.2 (3.7, 7.7)
In sub-Saharan Africa	317 (168, 496)	318 (168, 497)	175 (47.5, 320.8)
Onset location vs report location			
In same county	4,589 (53.0%)	4,554 (53.2%)	35 (35.7%)
In different counties of same province	3,625 (41.9%)	3,573 (41.8%)	52 (53.1%)
In different provinces	439 (5.1%)	428 (5.0%)	11 (11.2%)
Report location vs home/living location			
In same county	4,083 (47.2%)	4,056 (47.4%)	27 (27.6%)
In different counties of same province	3,477 (40.2%)	3,418 (40.0%)	59 (60.2%)
In different provinces	835 (9.6%)	824 (9.6%)	11 (11.2%)
Foreigner	258 (3.0%)	257 (3.0%)	1 (1.0%)
The admin level of first-visit health institution			
Province	898 (10.4%)	886 (10.4%)	12 (12.2%)
Prefecture	1,928 (22.3%)	1,915 (22.4%)	13 (13.2%)
County	3,227 (37.3%)	3,210 (37.5%)	17 (17.4%)
Township and lower	1,325 (15.3%)	1,297 (15.1%)	28 (28.6%)
Unknown	1,275 (14.7%)	1,247 (14.6%)	28 (28.6%)
The admin level of hospitals for final diagnosis and report			
Province	1,625 (18.8%)	1,588 (18.5%)	37 (37.8%)
Prefecture	3,044 (35.2%)	3,000 (35.1%)	44 (44.9%)
County	3,769 (43.5%)	3,752 (43.9%)	17 (17.3%)
Township and lower	215 (2.5%)	215 (2.5%)	0 (0)

Note: Data are presented as n (%) of patients unless otherwise indicated.

Supplementary Table S2. The community of origin-destination networks of *P. falciparum* malaria imported from SSA to mainland China, 2011-2015.

Community	Sub-Saharan country (n=41)	Province in mainland China (n=31)
1	Ghana (1)	Guangxi (1)
2	Sudan, Ethiopia, Sierra Leone, Togo, and Rwanda (5)	Xinjiang and Sichuan (2)
3	Angola, Equatorial Guinea, Republic of Congo, Gambia, Namibia, Zambia, South Africa, Zimbabwe, South Sudan, and Madagascar (10)	Qinghai, Ningxia, Henan, Jiangxi, Hebei, Anhui, Shandong, Jiangsu, and Jilin (9)
4	Senegal, Mauritania, Guinea Bissau, Guinea, Liberia, Mali, Ivory Coast, Burkina Faso, Niger, Benin, Nigeria, Gabon, Cameroon, Chad, Democratic Republic of the Congo, Central African Republic, Botswana, Burundi, Mozambique, Uganda, Malawi, Kenya, Eritrea, United Republic of Tanzania, and Somalia (25)	Beijing, Chongqing, Fujian, Gansu, Guangdong, Guizhou, Hainan, Heilongjiang, Hubei, Hunan, Liaoning, Inner Mongolia, Shanghai, Shanxi, Shaanxi, Tianjin, Tibet, Yunnan, and Zhejiang (19)

Note: The data in parentheses is the number of countries or provinces. The score of modularity is 0.219 with a resolution of 0.9.

Supplementary Table S3. ***Spearman*****'s rank correlation coefficients between the number of *P. falciparum* malaria cases and covariates.**

Covariate	Correlation coefficient (ρ)	P value
**1 Number of air passengers**	0.425	0.006
**2 *Pf*PR$_{2-10}$**	0.639	<0.001
**3 Total amount of ODA**	0.679	<0.001
**3.1 Resource extraction**	0.668	<0.001
- Energy generation and supply	0.629	<0.001
- Mining industry and construction	0.350	0.025
- Agriculture, forestry and fishing	0.542	<0.001
**3.2 Infrastructure**	0.475	0.002
- Transport and Storage	0.470	0.002
- Communications	0.361	0.020
- Utilities	0.373	0.016
**3.3 Health**	0.503	0.001
**3.4 Education**	0.303	0.054
**3.5 Multi-sector**	0.331	0.034
**3.6 Other**	0.393	0.011

Note: The analysis was based on the aggregated data by sub-Saharan country.

Supplementary Table S4. Performance of generalized linear models fitting the number of *P. falciparum* malaria cases by the amount of each ODA sector, adjusting for *Pf*PR_{2-10} and number of air passengers.

Formula in GLM models	With quasi-Poisson distribution function	
	R^2 in train (IQR)	R^2 in cross-validation (IQR)
Case ~ Passengers + *Pf*PR_{2-10} + ODA (total)	0.659 (0.633, 0.698)	0.577 (0.237, 0.797)
Case ~ Passengers + *Pf*PR_{2-10} + Resource	0.335 (0.314, 0.429)	0.303 (0.096, 0.654)
Case ~ Passengers + *Pf*PR_{2-10} + Infrastructure	0.171 (0.137, 0.208)	0.132 (0.021, 0.374)
Case ~ Passengers + *Pf*PR_{2-10} + Health	0.489 (0.455, 0.536)	0.116 (0.018, 0.507)
Case ~ Passengers + *Pf*PR_{2-10} + Education	0.310 (0.267,0.356)	0.110 (0.020, 0.478)
Case ~ Passengers + *Pf*PR_{2-10} + Multi-sector	0.493 (0.428, 0.622)	0.257 (0.051, 0.691)
Case ~ Passengers + *Pf*PR_{2-10} + Other	0.280 (0.220, 0.351)	0.056 (0.011, 0.269)

Note: The analysis based on the aggregated data by sub-Saharan country. In the cross-validation approach, 80% observations were randomly sampled as train set with remaining 20% as validation set, and the process was iterated 1000 times.

Supplementary Table S5. Factors associated with risk of death in *P. falciparum* malaria cases imported from sub-Saharan countries to mainland China between 2011 and 2015.

Risk factor	No (%) of fatal cases	Odds ratio (95% CI) of death			
		Crude OR	P value	Adjusted OR [a]	P value
**Gender**					
Female	92/8351 (1.10)	1.9 (0.7, 4.0)	0.153	2.0 (0.8, 4.4)	0.095
Male	6/302 (1.99)				
**Age**					
0-50 years	83/7969 (1.04)	2.1 (1.2, 3.6)	0.006	2.2 (1.2, 3.7)	0.007
>50 years	15/684 (2.19)				
**Nationality**					
Chinese	97/8395 (1.16)	2.6 (0.6, 60.8)	0.373	3.5 (0.8, 61,9)	0.218
Foreigner	1/258 (0.39)				
**Purpose of visit to sub-Saharan Africa**					
Labour service	91/7770 (1.17)	1.2 (0.6, 2.9)	0.588	1.2 (0.6, 3.1)	0.597
Other	7/738 (0.95)				
**Education**					
Primary or lower	30/1396 (2.15)	2.4 (1.5, 3.6)	<0.001	2.3 (1.5, 3.5)	<0.001
Secondary or higher	61/6611 (0.92)				
**Modularity of destination province**					
Community 1st	2/1548 (0.13)	Reference			
Community 2nd	14/597 (2.35)	17.4 (4.8, 121.2)	<0.001	18.2 (5.1, 116,4)	<0.001
Community 3rd	39/3422 (1.14)	8.3 (2.5, 54.9)	<0.001	8.7 (2.7, 53,7)	<0.001
Community 4th	43/3086 (1.39)	10.2 (3.1, 67.2)	<0.001	10.6 (3.3, 65,4)	<0.001

**GDP per capita of each province in 2015 (onset location)**					
<=12,000 US dollars	80/6293 (1.27)	1.7 (1.0, 2.9)	0.046	1.7 (1.1, 3.0)	0.036
>12,000 US dollars	18/2360 (0.76)				
**Month of onset**					
January - February	40/1788 (2.24)	2.7 (1.8, 4.0)	<0.001	2.7 (1.8, 4.0)	<0.001
March – December	58/6865 (0.84)				
**Duration from onset to diagnosis**					
>3 days	74/4163 (1.78)	3.4 (2.1, 5.4)	<0.001	3.4 (2,1, 5.4)	<0.001
<=3 days	24/4490 (0.53)				
**$PfPR_{2-10}$ in sub-Saharan countries in 2010-2015**					
<=20%	44/2863 (1.54)	1.7 (1.1, 2.5)	0.012	1.7 (1.1, 2.5)	0.013
>20%	54/5790 (0.93)				
**First-visit health institution**					
Township level or lower	28/1325 (2.11)	3.1 (1.9, 5.0)	<0.001	3.0 (1.9, 4.9)	<0.001
County level or higher	42/6053 (0.69)				

[a] The odds ratio was adjusted for gender, age and nationality if applicable.

Supplementary Table S6. The list of variables in the individual dataset of *P. falciparum* malaria cases imported from SSA to mainland China, 2011-2015.

Variables	Definition/classification	Completeness (N = 8,653)
Type of diagnosis	Clinical diagnosed or laboratory-confirmed case	100% reported
Age	The interval time from the date of birth to the date of onset	100% reported
Nationality	Chinese or foreigner	100% reported
Origin country	The country where the case infected with *P. falciparum* malaria	100% reported
Education	Illiteracy, Primary (6-year education), Junior secondary (9-year), Senior secondary (12-year), or Higher education (>12-year)	92.5% reported
Date of onset	The date of illness onset	100% reported
Date of diagnosis	The date of diagnosis as malaria	100% reported
Date of report	The date of report to surveillance system	100% reported
Date of Death	The date of case death, if applicable.	100% reported
Purpose of travel	Labour service or other	98.3% reported
Duration in Africa of Chinese cases	Days in Africa for Chinese cases	30.7% reported
Hospitalization	Inpatient or outpatient	97.3% reported
County of address	The address (county level) of case with illness onset	100% reported
County's code of address	A unique 8-digital number for each county	100% reported

Variables	Definition/classification	Completeness (N = 8,653)
Coordinates of address	Latitude and longitude of living address of case with illness onset	100% reported
Admin level of first-visit health institution	Province, prefecture, county, or township and lower	85.3% reported
First diagnosis as malaria	Yes, or No	86.2% reported
Admin level of hospitals for final diagnosis and report	Province, prefecture, county, or township and lower	100% reported
County's code of hospital	A unique 8-digital number for each county	100% reported
Coordinates of hospital	Latitude and longitude of address of hospital for final diagnosis and report	100% reported
Onset location vs. report (hospital) location	In same county, in different counties of same province, or in different provinces	100% reported
Report (hospital) location vs. home/living location	In same county, in different counties of same province, or in different provinces	100% reported

Supplementary Table S7. The amount of ODA by sector from China to sub-Saharan Africa between 2006 and 2013.

Sector and subsector	Number of projects	Million U.S. Dollars
**1 Resource extraction**	**263**	**28,566.06**
**1.1 Energy**	**99**	**17,541.91**
- Energy generation and supply	99	17,541.91
**1.2 Mining**	**46**	**7,245.99**
- Mining industry and construction	46	7,245.99
**1.3 Agriculture**	**118**	**3,778.16**
- Agriculture, forestry and fishing	118	3,778.16
**2 Infrastructure**	**319**	**35,732.79**
**2.1 Communications**	**72**	**4,619.94**
- Communications	72	4,619.94
**2.2 Transport**	**140**	**26,654.02**
- Transport and storage	140	26,654.02
**2.3 Utilities**	**107**	**4,458.83**
- Water supply and sanitation	36	2,889.33
- Other social infrastructure and services	71	1,569.50
**3 Education**	**206**	**961.53**
- Education	206	961.53
**4 Health**	**365**	**1,624.05**
- Health	351	1,612.13
- Women in development	9	10.52

Sector and subsector	Number of projects	Million U.S. Dollars
- Population policies/programmes and reproductive health	5	1.41
**5 Multi-sector**	**68**	**20,700.02**
- Other multisector	68	20,700.02
**6 Other**	**510**	**17,860.19**
- Unallocated / unspecified	179	7,595.64
- Banking and financial services	10	2,271.47
- Government and civil society	172	2,199.18
- General budget support	5	1,487.06
- Trade and tourism	29	1,486.67
- Action relating to debt	31	1,456.13
- Emergency response	41	1,267.40
- Developmental food aid/food security assistance	26	63.77
- Business and other Services	6	20.45
- Support to non-governmental organizations and government organizations	5	10.66
- General environmental protection	3	1.73
- Non-food commodity assistance	3	0.03
**Total**	**1731**	**105,444.64**

Note: The classification of subsector for each project was defined by the Development Assistance Committee of the Organization for Economic Cooperation and Development.[22] This study aggregated the subsector into sector (in bold). The monetary amount was deflated from reported currency to 2011 U.S. Dollars.

Supplementary Figures

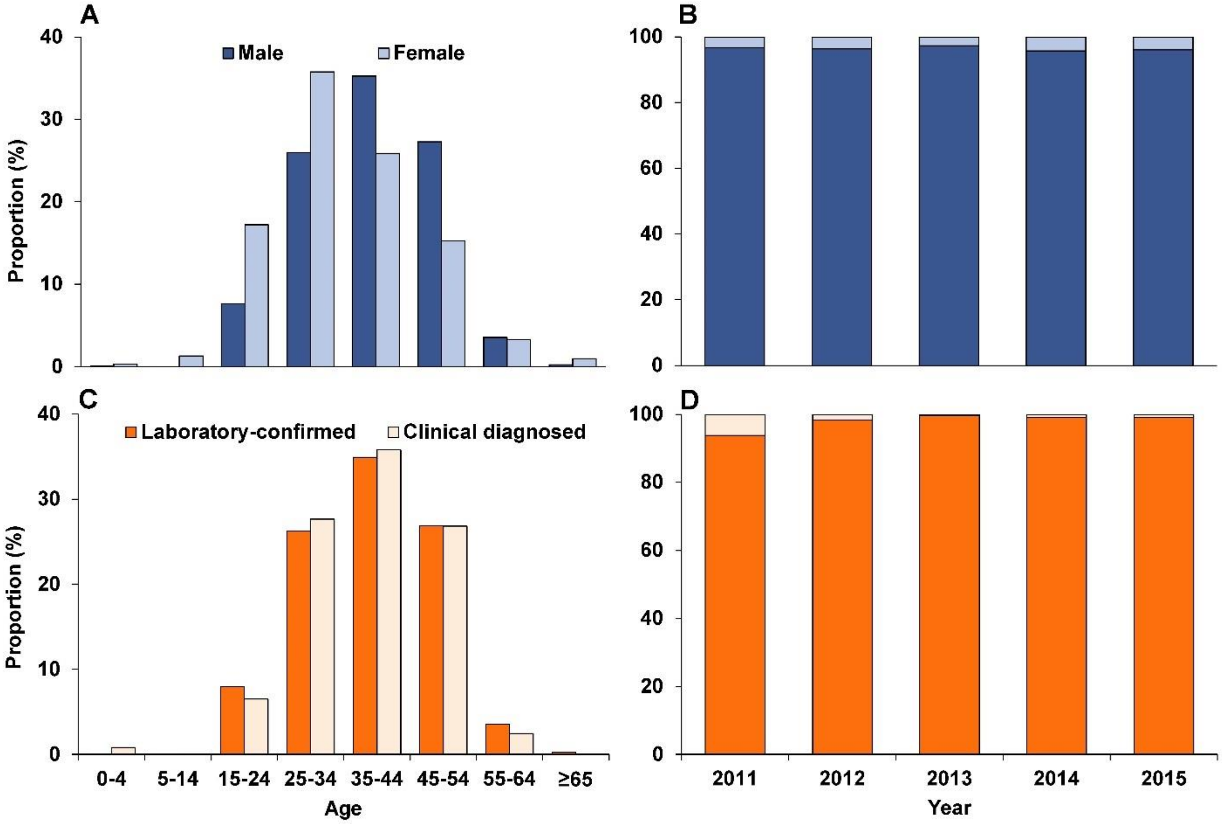

Supplementary Figure S1. Age distribution and proportion by different characteristics of *P. falciparum* malaria cases from sub-Saharan countries to provinces in mainland China, 2011-2015. (A) Age distribution of male and female cases. (B) Proportion of cases by sex each year. (C) Age distribution of laboratory-confirmed and clinical diagnosed cases. (D) Proportion of laboratory-confirmed and clinical diagnosed cases each year.

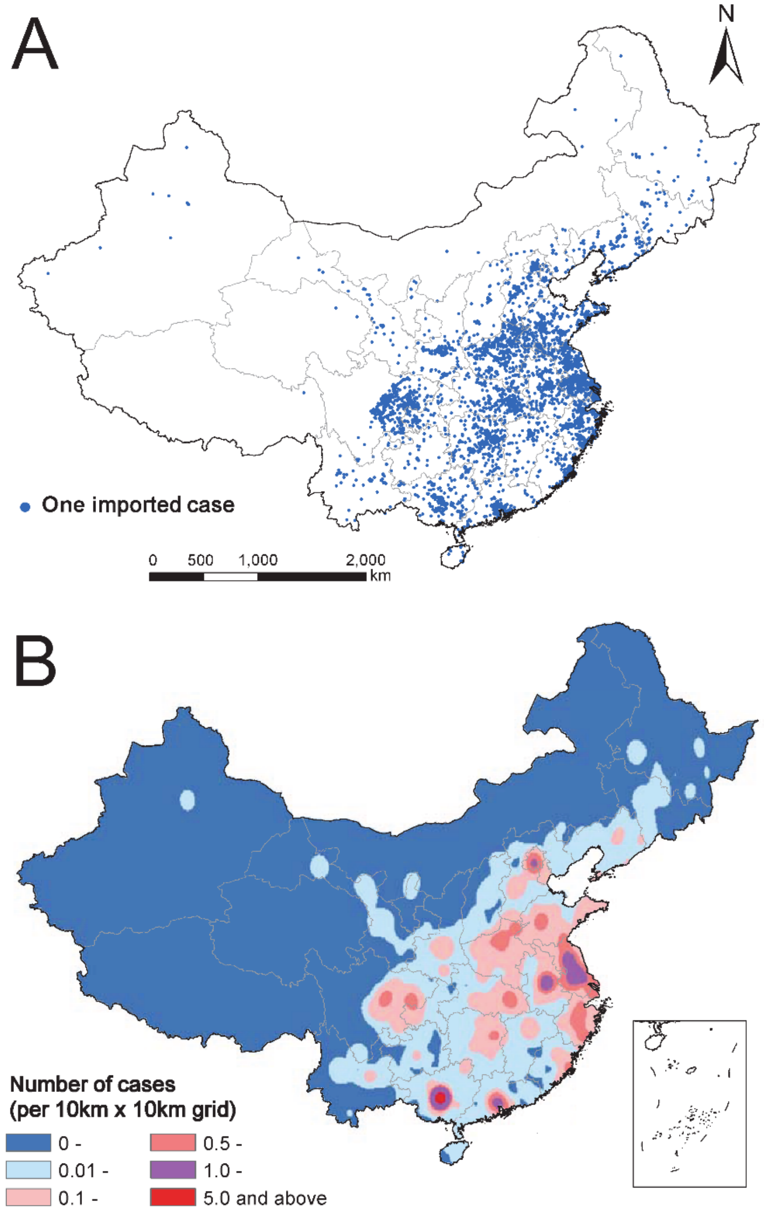

Supplementary Figure S2. Distribution of *P. falciparum* malaria cases in 31 provinces in mainland China imported from SSA, 2011-2015. (A) Geographic mapping of each by location with illness onset. (B) Density of cases per 10km^2 by kernel estimation. The centre of Guangxi province has the highest density (≥ 5 cases per 100 km^2), followed by Beijing, Jiangsu, Anhui and Guangdong provinces at eastern China (1.0 to 4.9 cases per 100km^2), and Sichuan province in western China and Henan province in central China (0.5 -0.9 cases per 100km^2). The map was created using ArcGIS 10.3 (http://www.esri.com/software/arcgis).

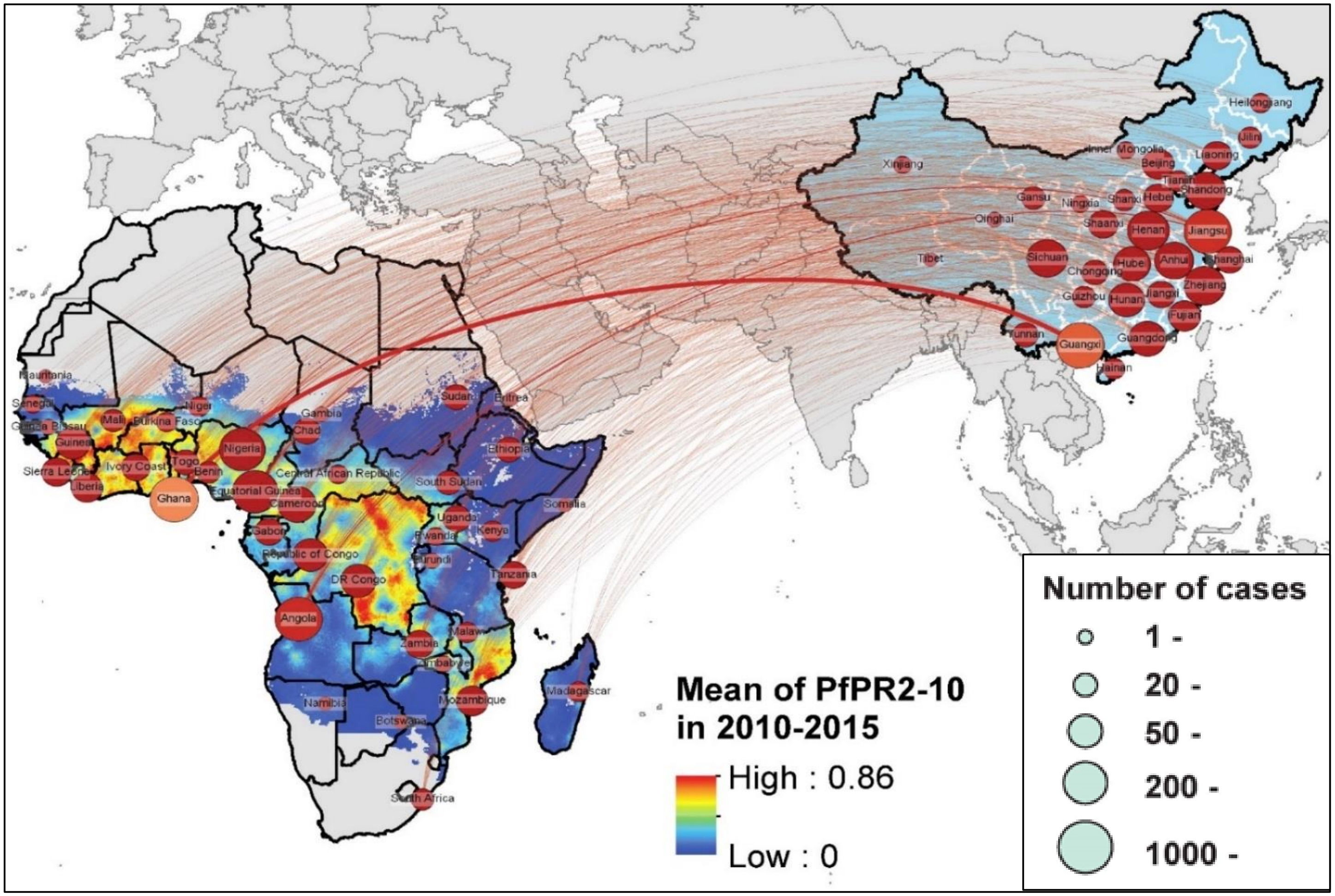

Supplementary Figure S3. Routes of *P. falciparum* malaria cases importation from sub-Saharan countries to provinces in mainland China, 2011-2015. Each line represents a pair of origin-destination, and the circle size and line weight represents the number of cases. The map was created using Gephi 0.8.2 (https://gephi.org/) and ArcGIS 10.3 (http://www.esri.com/software/arcgis).

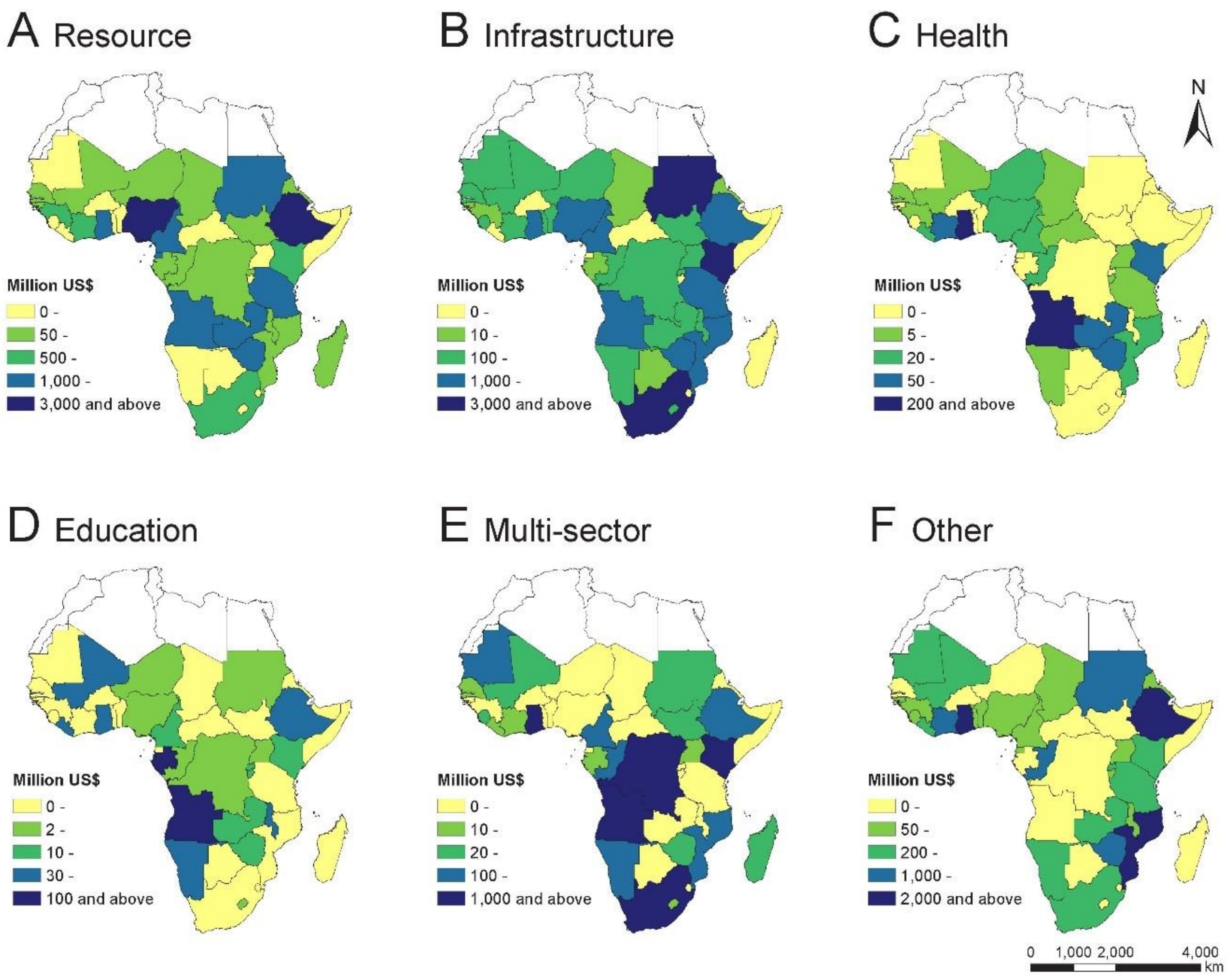

Supplementary Figure S4. The amount of ODA by sector from China to sub-Saharan countries between 2006 and 2013. The monetary amount of ODA was deflated from reported currency to 2011 U.S. Dollars. The map was created using ArcGIS 10.3 (http://www.esri.com/software/arcgis).

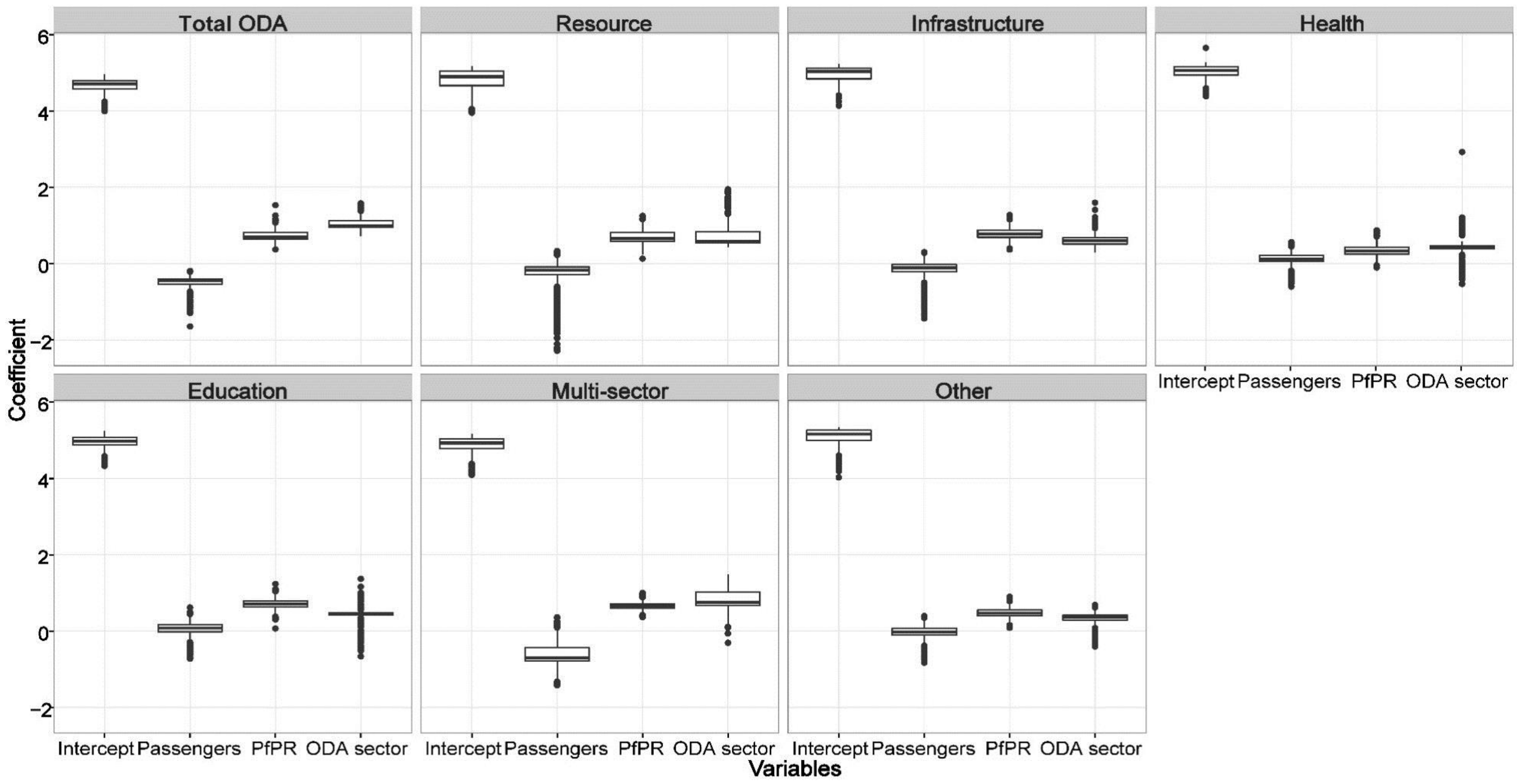

Supplementary Figure S5. Boxplot for coefficients of covariates in generalized linear models with quasi-Poisson distribution to fit the number of *P. falciparum* malaria cases by the amount of each ODA sector, adjusting for *Pf*PR$_{2\text{-}10}$ and number of air passengers. Each covariate was standardized, and the process of cross-validation was iterated 1000 times.

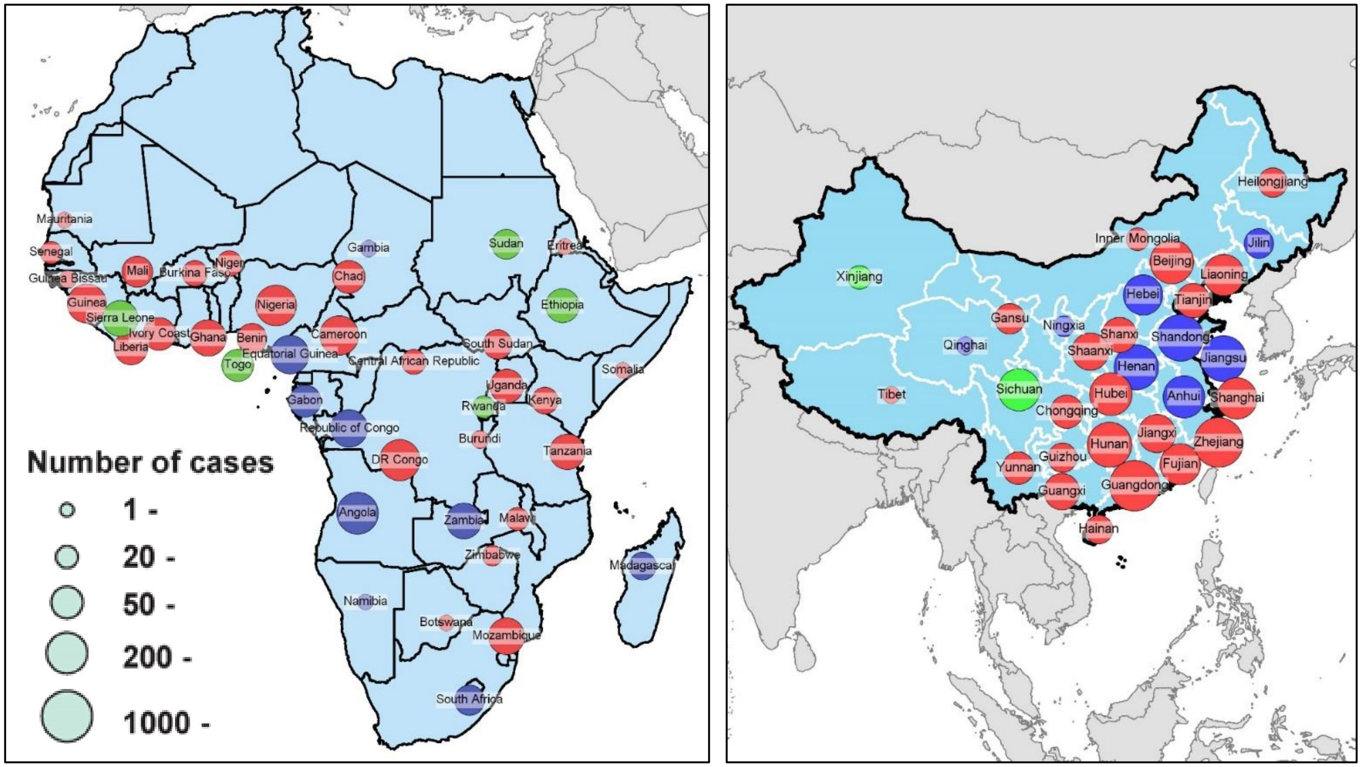

Supplementary Figure S6. Three communities of origin-destination networks of *P. falciparum* malaria cases from sub-Saharan countries to provinces in mainland China, 2011-2015, after removed the cases of Ghana-Guangxi pair in January - October, 2013 (N = 1,057). In 2013, Ghana began to strictly regulate the gold mining industry, which forced many Chinese gold miners to return to Guangxi province within a short time, and a substantial proportion of them (21.6%) infected with malaria.[14] We therefore exploited the communities of networks again after removing the cases returned from Ghana during this special event. The same colour of circles represents the same community of origin-destination networks detected by modularity analysis. The score of modularity is 0.206 with a resolution of 1.1. The map was created using Gephi 0.8.2 (https://gephi.org/) and ArcGIS 10.3 (http://www.esri.com/software/arcgis).

References

1 National Health and Family Planning Commission of the People's Republic of China. *Diagnostic criteria for malaria (WS 259-2006)* http://www.nhfpc.gov.cn/zwgkzt/s9499/201410/d29f0a078dd143f8b6374ed23dc40400.shtml (2006)

2 Sun, J.-L. *et al.* Comparative evaluation of the diagnosis, reporting and investigation of malaria cases in China, 2005–2014: transition from control to elimination for the national malaria programme. *Infectious Diseases of Poverty* **5**, 1-10 (2016).

3 Bhatt, S. *et al.* The effect of malaria control on Plasmodium falciparum in Africa between 2000 and 2015. *Nature* **526**, 207-211 (2015).

4 Huang, Z. & Tatem, A. J. Global malaria connectivity through air travel. *Malar. J.* **12**, 269 (2013).

5 Huang, Z., Wu, X., Garcia, A. J., Fik, T. J. & Tatem, A. J. An open-access modeled passenger flow matrix for the global air network in 2010. *PLoS One* **8**, e64317 (2013).

6 Strange, A. M., Dreher, A., Fuchs, A., Parks, B. & Tierney, M. J. Tracking Underreported Financial Flows: China's Development Finance and the Aid–Conflict Nexus Revisited. *Journal of Conflict Resolution* (2015).

7 Strange, A. M. P., B.; Perla, C.; Desai, H. *AidData's methodology for tracking underreported financial flows (Version 1.2)* http://china.aiddata.org/TUFF_codebook (2015).

8 National Bureau of Statistics of China. *Statistical Communiqué of the People's Republic of China on the 2015 National Economic and Social Development* http://www.stats.gov.cn/tjsj/zxfb/201602/t20160229_1323991.html (2016

9 National Bureau of Statistics of China. *National Data* http://data.stats.gov.cn/english/index.htm (2016)

10 Silverman, B. W. *Density estimation for statistics and data analysis*. (Chapman and Hall, 1986).

11 Vincent, D. B., Jean-Loup, G., Renaud, L. & Etienne, L. Fast unfolding of communities in large networks. *Journal of Statistical Mechanics: Theory and Experiment* **2008**, P10008 (2008).

12 Newman, M. E. Analysis of weighted networks. *Phys. Rev. E. Stat. Nonlin. Soft. Matter. Phys.* **70**, 056131 (2004).

13 Lambiotte, R., Delvenne, J. C. & Barahona, M. Random Walks, Markov Processes and the Multiscale Modular Organization of Complex Networks. *IEEE Transactions on Network Science and Engineering* **1**, 76-90 (2014).

14 Li, Z. *et al.* Malaria imported from Ghana by returning gold miners, China, 2013. *Emerg. Infect. Dis.* **21**, 864-867 (2015).

15 Lehman, A. *Jmp For Basic Univariate And Multivariate Statistics: A Step-by-step Guide.* (SAS Press, 2005).

16 Stigler, S. M. Francis Galton's Account of the Invention of Correlation. *Statistical Science* **4**, 73-79 (1989).

17 Fieller, E. C., Hartley, H. O. & Pearson, E. S. Tests for rank correlation coefficients. I. *Biometrika* **44**, 470-481 (1957).

18 WEDDERBURN, R. W. M. Quasi-likelihood functions, generalized linear models, and the Gauss—Newton method. *Biometrika* **61**, 439-447 (1974).

19 Enki, D. G. *et al.* Automated biosurveillance data from England and Wales, 1991-2011. *Emerg. Infect. Dis.* **19**, 35-42 (2013).

20 Tatem, A. J. *et al.* Air travel and vector-borne disease movement. *Parasitology* **139**, 1816-1830 (2012).

21 Lukacs, E. A Characterization of the Normal Distribution. *The Annals of Mathematical Statistics* **13**, 91-93 (1942).

22 Organization for Economic Cooperation and Development. *Purpose Codes: sector classification* http://www.oecd.org/dac/stats/purposecodessectorclassification.htm (2016)
